# A Potential Renewed Use of Very Heavy Ions for Therapy: Neon Minibeam Radiation Therapy

**DOI:** 10.3390/cancers13061356

**Published:** 2021-03-17

**Authors:** Yolanda Prezado, Ryochi Hirayama, Naruhiro Matsufuji, Taku Inaniwa, Immaculada Martínez-Rovira, Olivier Seksek, Annaïg Bertho, Sachiko Koike, Dalila Labiod, Frederic Pouzoulet, Laura Polledo, Nils Warfving, Aléthéa Liens, Judith Bergs, Takashi Shimokawa

**Affiliations:** 1 Institut Curie, Université PSL, CNRS UMR3347, Inserm U1021, Signalisation Radiobiologie et Cancer, 91400 Orsay, France; annaig.bertho@curie.fr; 2 Université Paris-Saclay, CNRS UMR3347, Inserm U1021, Signalisation Radiobiologie et Cancer, 91400 Orsay, France; 3 Department of Charged Particle Therapy Research, National Institute of Radiological Sciences (NIRS), National Institutes for Quantum and Radiological Science and Technology, Chiba 263-8555, Japan; hirayama.ryoichi@qst.go.jp (R.H.); matsufuji.naruhiro@qst.go.jp (N.M.); inaniwa.taku@qst.go.jp (T.I.); koike.sachiko@qst.go.jp (S.K.); shimokawa.takashi@qst.go.jp (T.S.); 4 Department of Accelerator and Medical Physics, National Institute of Radiological Sciences (NIRS), National Institutes for Quantum and Radiological Science and Technology, Chiba 263-8555, Japan; 5 Ionizing Radiation Research Group, Physics Department, Universitat Autònoma de Barcelona (UAB), E-08193 Cerdanyola del Vallès, Spain; immamartinez@gmail.com; 6 Université Paris-Saclay, CNRS/IN2P3, Université de Paris, IJCLab, Pole Santé, 91405 Orsay, France; seksek@ijclab.in2p3.fr; 7 Experimental Radiotherapy Platform, Translational Research Department, Institut Curie, Université Paris Saclay, 91400 Orsay, France; dalila.labiod@curie.fr (D.L.); frederic.pouzoulet@curie.fr (F.P.); 8 AnaPath Services GmbH, Hammerstrasse 49, 4410 Liestal, Switzerland; lpolledo@anapath.ch (L.P.); nwarfving@anapath.ch (N.W.); aliens@anapath.ch (A.L.); 9 Department of Radiology Charité—Universitätsmedizin Berlin, CCM Charitéplatz 1, 10117 Berlin, Germany; jbergs79@gmail.com

**Keywords:** minibeam radiation therapy, heavy ion therapy, hypoxic tumors, normal tissue toxicity

## Abstract

**Simple Summary:**

The treatment of hypoxic tumours continues to be one of the main challenges for radiation therapy. Minibeam radiation therapy (MBRT) shows a highly promising reduction of to-xicity in normal tissue, so that very heavy ions, such as Neon (Ne) or Argon (Ar), with extremely high LET, might become applicable to clinical situations. The high LET in the target would be unrivalled to overcome hypoxia, while MBRT might limit the side effects normally preventing the use of those heavy ions in a conventional radiotherapeutic setting. The work reported in this manuscript is the first experimental proof of the remarkable reduction of normal tissue (skin) toxicities after Ne MBRT irradiations as compared to conventional Ne irradiations. This result might allow for a renewed use of very heavy ions for cancer therapy.

**Abstract:**

(1) Background: among all types of radiation, very heavy ions, such as Neon (Ne) or Argon (Ar), are the optimum candidates for hypoxic tumor treatments due to their reduced oxygen enhancement effect. However, their pioneering clinical use in the 1970s was halted due to severe side effects. The aim of this work was to provide a first proof that the combination of very heavy ions with minibeam radiation therapy leads to a minimization of toxicities and, thus, opening the door for a renewed use of heavy ions for therapy; (2) Methods: mouse legs were irradiated with either Ne MBRT or Ne broad beams at the same average dose. Skin toxicity was scored for a period of four weeks. Histopathology evaluations were carried out at the end of the study; (3) Results: a significant difference in toxicity was observed between the two irradiated groups. While severe da-mage, including necrosis, was observed in the broad beam group, only light to mild erythema was present in the MBRT group; (4) Conclusion: Ne MBRT is significantly better tolerated than conventional broad beam irradiations.

## 1. Introduction

The treatment of radioresistant hypoxic tumours is still one of the major challenges in radiation therapy [1]. Accumulating evidence indicates that hypoxia is responsible for inducing radiation and drug resistance [2] and it raises the likelihood of distant metastases [3]. Because of their increased linear energy transfer, heavy ions, like Neon, Silicon, or Argon, provide a reduced oxygen enhancement effect [4]. Linked to that, evidence exists that (very) heavy ions are more advantageous than X-rays (conventional radiation therapy) for the treatment of those hypoxic and radioresistant tumours [5,6]. Pioneering radiobiological evaluations showed that resistant cells of hypoxic tumours could be effectively killed with heavy ions beams [6], such as Silicon and Argon. However, the late adverse side effects that were observed in the few patients treated with those ions between 1979 and 1982 halted the use of those particles for therapy [6]. 

The remarkable reduction of normal tissue toxicities observed after both X-rays and proton minibeam radiation therapy (MBRT) as compared to conventional broad beam irradiations [7,8,9,10,11,12,13] might allow an improved therapeutic ratio. 

MBRT uses narrow beams (0.5 to 1 mm) to create a distinct spatial modulation of the dose, featuring alternating regions of high dose (peaks) and low dose (valleys) [14]. MBRT with both x-rays and protons was shown to provide a remarkable normal (skin and brain) tissue preservation in preclinical experiments [7,9,10,11], including the sparing of cognitive, emotional, and motor processes [12]. Average doses from 25 to 30 Gy, with peak doses up to 70 Gy, in one fraction, proved to be well tolerated [10,11,12,15]. In addition, at the same level of those tolerable doses in MBRT, the latter resulted in an equivalent, or even superior (glioma), tumour control as compared to conventional irradiation in small animal experiments [8,16,17,18,19,20]. A significant increase of lifespan was achieved, even with highly he-terogeneous dose distributions in the tumour and large areas of the tumour only covered by doses as low as 5.8 Gy [15,17,19]. Consequently, MBRT has a high potential to widen the therapeutic index for radioresistant tumours’ treatment.

A further improvement could be to use (very) heavy ions (Ne, Si, Ar), thanks to their superior relative biological effectiveness and reduced oxygen effect as compared to X-rays and protons, provided that normal tissue toxicity stays below the tolerance limit. Advantageous dose distributions for normal tissue sparing were obtained in our recent Monte Carlo (MC) studies [21,22] on heavy ions MBRT: extremely high peak-to-valley dose ratio (PVDR) values (>100), very narrow penumbras, and low valley doses were achieved in the first centimetres as well as in the fragmentation tail, helping in the sparing of proximal normal tissues. Although the yield of secondary nuclear products increases with atomic number, the actual dose being deposited by the secondary nuclear fragments in the valleys only becomes dominant at the Bragg peak (tumour) position [21]. These results supported the further exploration of this avenue.

We performed the first in vivo evaluation of very heavy ions MBRT to confirm our hypothesis. In particular, we chose to start with Ne ions. since our Monte Carlo calculations indicated that, among all heavy ions, they offer the best balance between having a high peak-to-valley dose ratio and peak-to-valley-linear energy transfer ratio in normal tissues and high linear energy transfer values (close to 100 keV/µm) in the target region [22]. 

## 2. Materials and Methods

### 2.1. Irradiations

**Figure 1:**
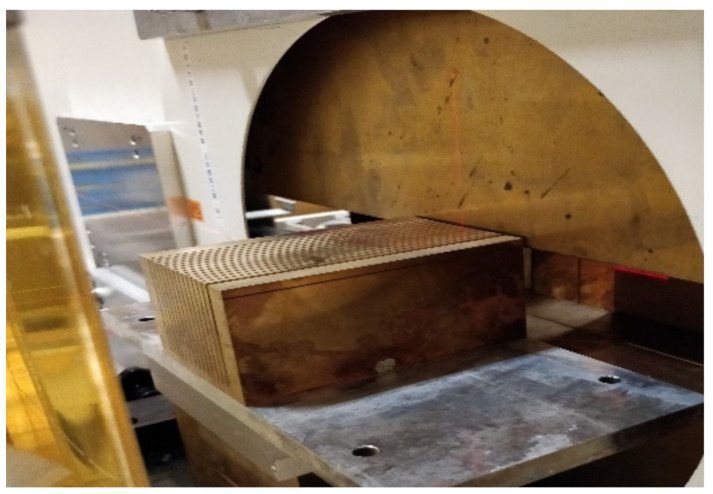
Photograph of the multislit brass collimator placed at the jaws exit of the beamline at biology room of HIMAC.

The irradiations were carried out in the biology room at Heavy Ion Medial Accelerator (HIMAC) of the National Institute of Radiological Sciences, Chiba, Japan [23]. A beam of 230 MeV/u (45 keV/µm [24]) Ne ions was used. Ne minibeams were generated by means of a custom-made 10 cm-thick brass collimator. The latter has slit widths and centre-to-centre distances of 700 µm and 3500 µm, respectively, see Figure 1. The total irradiation field size was 4.5 × 4.5 cm^2^. The dose rate was 2 Gy/min.

**Figure 2:**
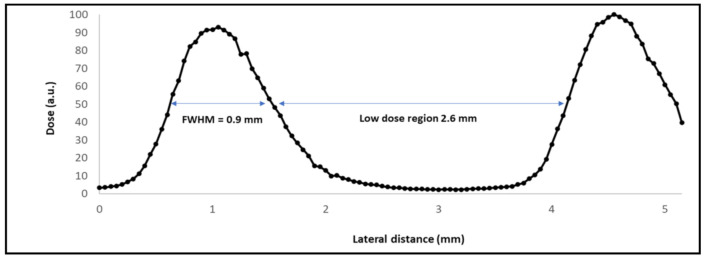
Central part of a minibeam radiation therapy (MBRT) dose profile measured at 5 mm depth in water by means of the microdiamond detector.

Dosimetry was performed using a two-step protocol, which was similar to previous works [14]. Absolute dosimetry was carried out in broad beam conditions (4.5 cm^2^ field size) with an ionisation chamber (PTW Markus chamber). The relative dosimetry was realised by means of two high spatial resolution detectors: a PTW 60019 microdiamond detector [25] and gafchromic films (for crosscheck). Figure 2 shows the central part of a dose profile that was measured with the microdiamond detector at 5 mm depth in water. The full width half at maximum (FWHM) of the high dose (peak) and low dose regions are 0.9 mm and 2.6 mm, respectively. 

**Figure 3:**
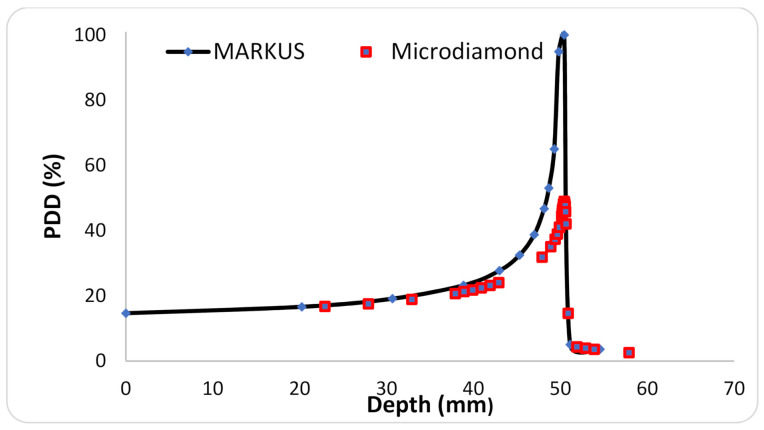
Percentage depth dose curves (PDD) of a 230 MeV/u Ne beam measured with a Markus ionisation chamber (blue points) and with the PTW microdiamond detector (red squares).

The PTW microdiamond was cross-calibrated in the Ne beam towards the Markus ionisation chamber in broad beam conditions. Subsequently, the possible impact of quenching effects in Ne ion beams was assessed. The quenching effects are caused by a local (microscopic) saturation, which occurs around highly ionizing ion tracks. The result is a reduction of the dose actually measured by the detector. The effect depends on the linear energy transfer (LET) of the radiation, and it has been reported in films and microdiamond detectors exposed to heavy ions [26,27]. The quenching effect was assessed by comparing the depth curves that were measured with the microdiamond and the Markus chamber. See Figure 3. A 50% quenching effect was estimated at the Bragg peak position. No quenching effect was observed in the plateau region (normal tissues), which is the area where the legs were going to be irradiated. 

A large area chamber (IBA, 12 cm diameter) was also used to evaluate the scatter factors, as in previous works [28].

### 2.2. In Vivo Experiment

Ethics statement: all animal experiments were conducted in accordance with the animal welfare and ethical guidelines of our institutions. They were approved by the National Institute of Radiological Sciences Institutional Animal Care and Use Committee (permit no. 19–2004). 

The legs of C57BL/6J mice (female, eight weeks old) were irradiated and the skin response was evaluated in order to assess the potential reduction of normal tissue toxicity of Ne MBRT as compared to broad beam irradiations. The mice were anaesthetised for irradiation with pentobarbital Sodium Salt (~0.5 mg/10 g body weight). 

Two groups of animals were considered: (i) one group received broad beam (BB) conventional irradiation (20 ± 1 Gy, N = 8); (ii) a second group received Ne MBRT with the same mean dose as the BB group (mean dose 20 ± 2 Gy, peak dose 60 ± 6 Gy, valley dose 1.1 ± 0.1 Gy, N = 8). Non-irradiated legs served as controls. The doses reported here are physical doses and they were delivered in one fraction. Relative biological effectiveness (RBE) values of between 1.5 and 2 have been previously reported in the plateau region of Ne beams [29,30]. Therefore, although the RBE concept might not be directly applicable for MBRT, a similar biological effect as a single-fraction irradiation of at least 40 Gy with X-rays could be expected. 

**Figure 4:**
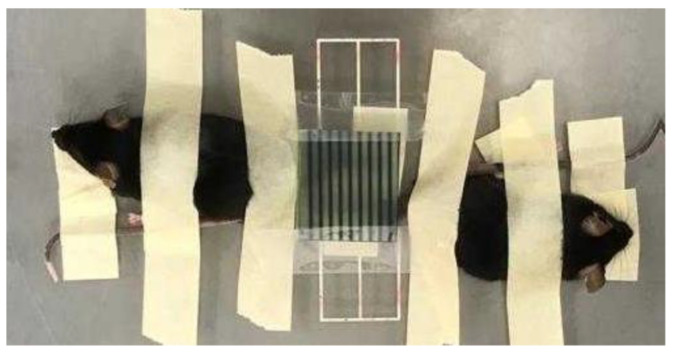
Irradiation setup: the gachromic film shows the pattern of the irradiation.

Gafchromic films were placed on top of the legs to assess the quality of the irradiations, see Figure 4. The films corroborated the profile in Figure 2. 

The animals were followed up for four weeks. The skin reaction was scored every two to three days using the arbitrary score that is shown in Table 1, which is inspired by previous work on mouse skin irradiations [31]. 

At the end of the study the animals were sacrificed, and the legs were cut and immersed in formol 10%. After 24 h, the legs were embedded in paraffin; 4-μm-thick sections were cut and stained in haematoxylin and eosin (HE). All the samples were image scanned by an Olympus Slideview VS200 slides scanner using an Olympus U-TV1XC camera and 20× objective. The histopathological (double-blinded) evaluation was carried out by board certified pathologists (European college of veterinary pathologists (ECVP)). Histological changes were described according to the distribution, severity, and morphologic character. The severity scores were assigned grade 1 to 5, as described in Appendix A. 

## 3. Results

**Figure 5:**
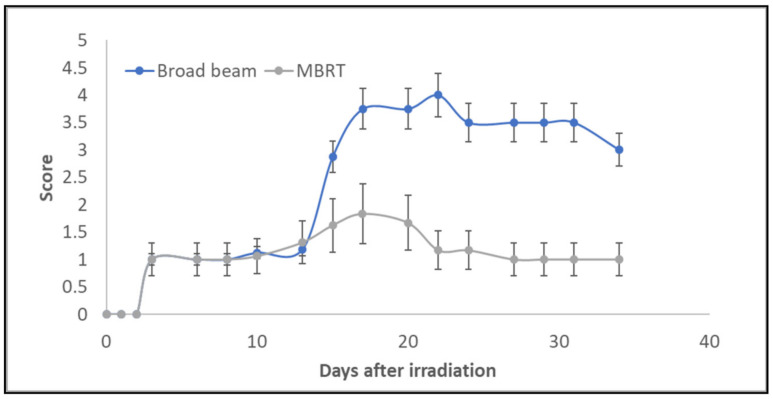
Evaluation of average skin damage scores as a function of follow up time and irradiation mode. Mean and standard deviation values are represented in the plot.

**Figure 6:**
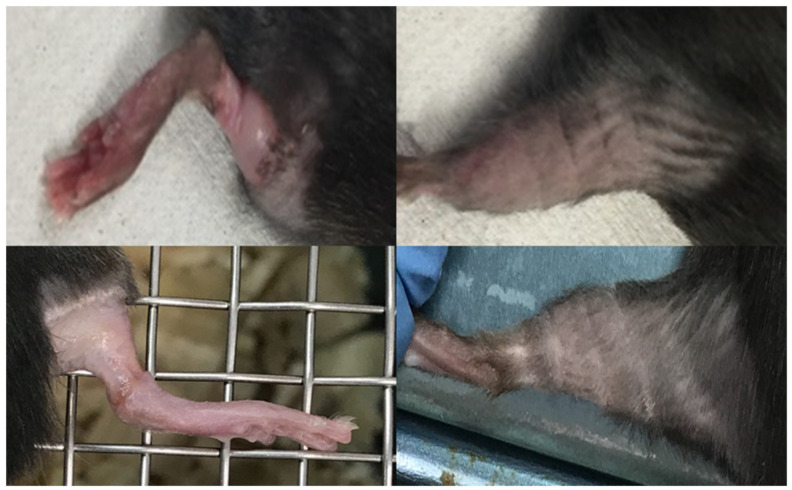
Photographs of skin responses. Upper row: skin response two weeks after irradiation. Left: mouse’s leg irradiated with Ne broad beam, showing moist desquamation and ulceration. Right: leg of mouse two weeks after receiving Ne MBRT, exhibiting erythema. Lower row: mice’s legs four weeks after irradiation. Left: mouse’s leg irradiated with Ne broad beam, exhibiting extensive damage. Right: leg of one mouse in the MBRT group showing how the skin has almost recovered.

The irradiated animals gained weight at the same rate as the controls. Figure 5 shows the evolution of the skin damage scores (according to Table 1) for the two irradiated groups over time. A net difference was observed between the broad beam and MBRT groups from the 14th day after irradiation. Macroscopically, broad beam irradiated skin showed a focally extensive area of hair loss with cutaneous erosion/ulceration of appro-ximately 4.7 mm-length, on average. In contrast, skin irradiated with MBRT presented multifocal, equidistant, parallel, < 1 mm thick lines of hair loss and erythema without ulceration, see Figure 6. Two weeks after irradiation, six out of eight animals receiving broad beam irradiation presented extensive damage with a skin damage score that reached up to four and were sacrificed. Mice that were treated with MRBT survived the whole study period. A maximum score of 1.8 was reached in the MBRT group, 17 days after irradiation. From day 20, only light erythema was observed in this group. 

**Figure 7:**
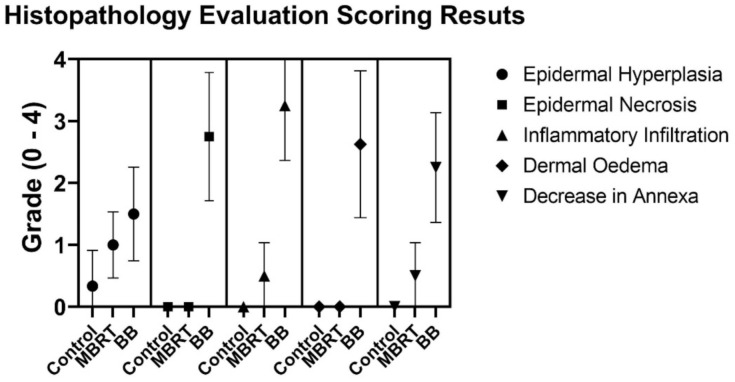
Histology evaluation scoring results showing mean values and standard deviations for the different irradiation modes.

Table 2 and Figure 7 summarize the scores from the histology evaluation in each treatment group. Individual data from the histopathology evaluation per animal are presented in a table in the Supplemental Material (Table S1). 

**Figure 8:**
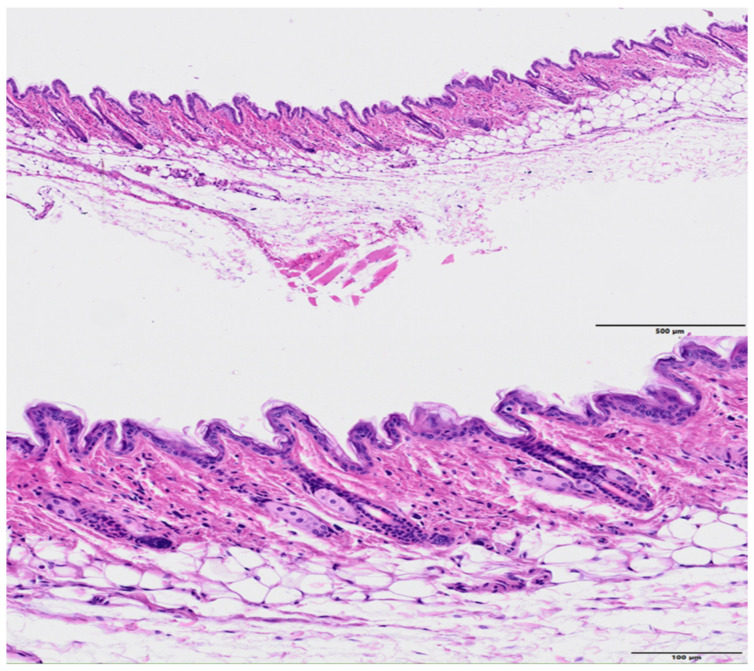
Histology images of a control (non-irradiated leg), HE. Up: no abnormal histopathological findings were observed (bar = 500 µm). Low: higher magnification (bar = 100 µm) showing the normal histological cutaneous structure.

The skin from control (non-irradiated) legs did not show any relevant abnormalities, see Figure 8.

**Figure 9:**
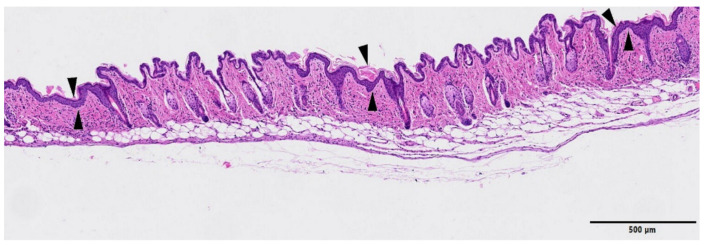
MBRT irradiation. HE staining, Bar = 500 µm. Three multifocal areas of epidermal hy-perplasia (minimal) are present in the image (arrowheads), where the epidermal layer appears thickened and the subjacent dermis showed lack of annexal structures. On the subjacent corresponding areas, there was a decrease in the number of hair follicles, apocrine glands, and sebaceous glands. There was also a minimal and multifocal in-flammatory infiltrate.

**Figure 10:**
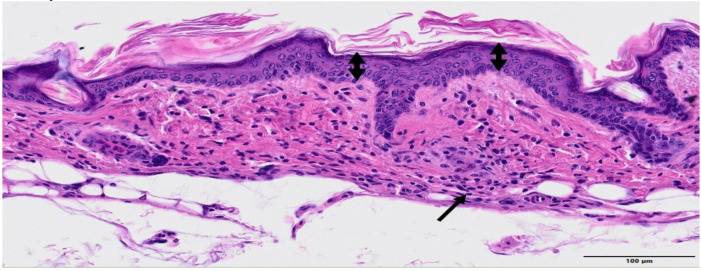
MBRT irradiation. HE staining. Higher magnification. Bar = 100 µm. Higher magnification that shows the epidermal focal increase from 1–2 layers (left and right edges) to 4–5 multiple layers (minimal epidermal hyperplasia, double-headed arrow). The subepidermal presence of small clusters of inflammatory cells (arrow).

The skin exposed to MBRT irradiation presented multifocal epidermal hyperplasia in low severity, which was characterized by an increase in thickness of the epidermal layer from single-cell layer to 2–3 multilayers. Often, there were minimal infiltrates of inflammatory cells and/or decrease in the presence of annexal structures (hair follicles, sebaceous glands, and apocrine glands), see Figure 9 and Figure 10 (higher magnification). The lesions extended over an average length of 487 µm and they were spaced by 1.1 mm, on average. This pattern does not seem to follow the peak and valley dose profiles delivered and shown in Figure 2.

**Figure 11:**
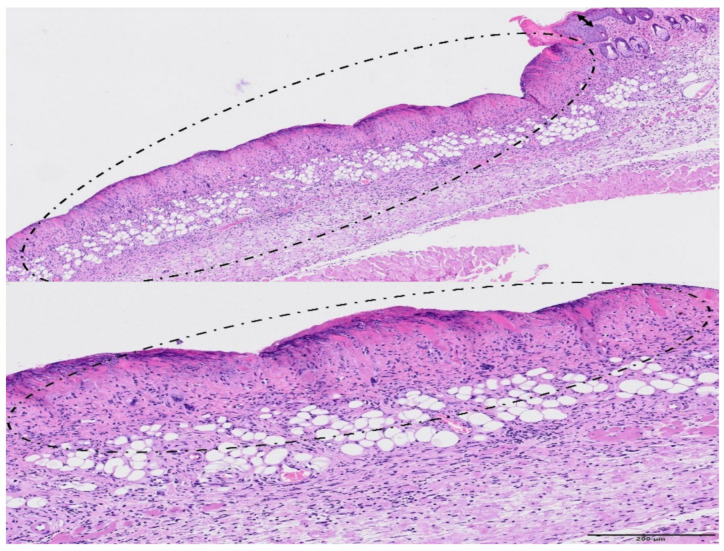
Broad beam irradiation. HE staining. Upper figure (bar = 500 µm): a large area of the skin presented extensive epidermal necrosis (encircled), with abundant inflammatory infiltration and loss of annexa. There was also epidermal hyperplasia in less affected areas (double-headed arrow). Lower figure—Bar = 200 µm: note that the loss of cellular detail of the epidermis (epidermal necrosis, encircled) at higher magnification. The subjacent dermis appears highly infiltrated by inflammatory cells.

**Figure 12:**
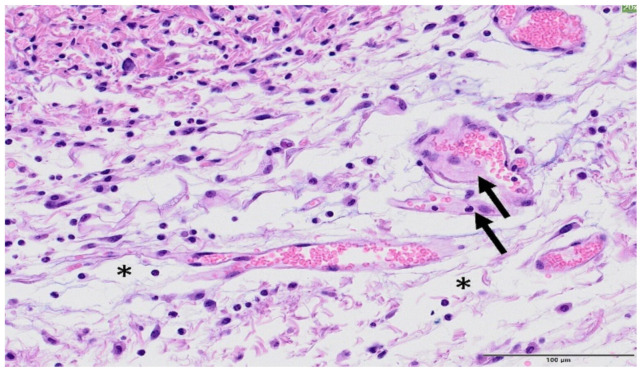
Broad beam irradiation. HE staining, Bar = 100 µm. Within the dermis, there is an inflammatory infiltrate consisting of macrophages, lymphocytes, and neutrophils. The collagen fibres from the connective tissue were separated by oedema (white spaces, asterisk). In the image, a couple of vessels present early hyalinization (arrows).

The skin that is irradiated with broad beam presented relevant changes of epidermis and dermis, epidermal necrosis, associated with inflammation, oedema, and loss of the annexal structures, see Figure 11. There was a focally extensive loss of epidermis and dermis (ulceration), with replacement by abundant eosinophilic cellular and karyorrhectic debris and an overlying serocellular crust. The multifocal loss of the annexal structures was often detected. Blood vessels subjacent to the necrosis presented reactive endothelium and they were often hyalinized or contained fibrin thrombi. There was an inflammatory infiltrate in the adjacent dermis with numerous granulocytes, macrophages, lymphocytes, and fewer plasma cells. see Figure 12. The connective tissue from the dermis also presented oedema, haemorrhage, and reactive fibroblasts. Epidermal hyperplasia was also often detected in less affected areas, often in the edges of the ulceration. The onset of severe damage occurred significantly earlier than the one reported in previous studies with X-rays at 40 and 80 Gy in one fraction [31]: two weeks in Ne BB (20 Gy) vs. 21 days with X-rays.

## 4. Discussion

Despite major advances in RT in the last decades, the treatment of hypoxic tumours remains elusive. In this context, the use of very heavy ions for therapy (e.g., Ne, Si, Ar), which are less dependent on the oxygen effect than X-rays, was explored in the past (BEVALAC facility, Berkeley, CA USA). Between 1975 and 1992, 433 patients were treated with C, N, O, Ne, Si, and Ar ions [6] for various malignancies. Very heavy ions (i.e., Ne) showed encouraging results in terms of local control of hypoxic tumours, for the treatment of macroscopic salivary gland carcinomas, paranasal sinus tumours, soft tissue sarcomas, macroscopic sarcomas of bone, locally advanced prostate carcinomas, and biliary tract carcinomas [32]. Unfortunately, serious late damage, including fatal complications, were reported [6]. For that reason, the use of such particles for therapy was rapidly discontinued, a few years after their first use. 

The work that is presented in this paper aims to explore a new RT approach that might allow for a renewed and optimal use of such very heavy ions for therapy. In this first evaluation, we were able to demonstrate that the alliance of MBRT and Ne ions leads to a net reduction of toxicity as compared to conventional broad beam irradiations in the plateau region (normal tissues). In our evaluation, severe toxicity was observed after conventional Ne broad beam irradiations, including radionecrosis. The onset of severe damage was significantly earlier (14 days versus 21 days) than higher doses (40 and 80 Gy) deposited in X-ray irradiations (28). In contrast, only light to mild erythema was present after MBRT, despite peak doses of 60 Gy (120 Gy biological equivalent dose, approximately) delivered in one sole fraction. Additionally, the damage seemed reversible, as reflected by the reduction of the damage score from the 20th day after irradiation. Thus, the damage may be considered adaptive and of low clinical significance. 

It should also be highlighted that the peak dose deposited in this experiment (60 Gy) is significantly higher than the ones employed in previous skin irradiation experiments while using conventional (seamless) exposures and that led to severe skin toxicity. In particular, doses higher than 24 Gy (X-rays) in one fraction have been previously reported to lead to a high risk for severe and irreversible skin toxicity [33]. A dose of 25.9 Gy delivered with 30 MeV protons induced moist desquamation (score 3 in our study) in 50% of the irradiated mice [34]. Along this line, heavy ions are even more effective than X-rays and protons inducing skin damage: doses between 20 Gy (80 keV/µm) and 30 Gy (14 keV/µm) of carbon ions resulted in 25% skin shrinkage [35]. Additionally, a dose of 18 Gy (one fraction) of Ne ions (80 keV/um) delivered to the mice’s feet induced the same isoeffect than 50 Gy delivered with gamma rays (red foot or dry desquamation in 150% of the area with epilation 250% of the area) [36]. Therefore, we can conclude than MBRT offers a net gain in normal (skin) tissue sparing. 

It is also worth noting that the average length (487 µm) and spacing (1.1 mm) among lesions do not directly correspond to the lengths of the peak and valley dose regions. Consequently, this disregards a simple dose effect. Instead, the pattern of the lesions points to a more global or collective tissular effect. It might be due “beneficial” bystander effects leading to tissue restoration, already observed in previous studies in skin [37,38]. Those effects could be initiated by strong cell-signalling cascades and cytokines release after cell death in the high dose regions [39]. Interestingly, the length of areas without lesion (1.1 mm in average) corresponds well to the ones that were found in in-depth evaluations of spatiotemporal proliferation and migration during wound healing in mouse skin epidermis [40].

The minimal toxicity observed after Ne MBRT irradiations is coherent with the results of our previous theoretical works [21,22], which predicted favourable dose distributions, with very low valleys in normal tissues and a low impact of nuclear fragments with high linear energy transfer. Concerning the fragmentation tail, our previous works [21] showed that spatially fractionation of the dose (and, thus, its tissue preservation) is also maintained at the tail region. Indeed, low valley doses were also obtained in the tail region. Consequently, a good tissue sparing could be expected in that region. 

The gain in normal tissue preservation opens the door to a potential renewed use of those very heavy ions for therapy. Several works have reported an equivalent or superior tumour control with both x-rays and protons MBRT than with BB [15,17,18,19]. This outcome has been obtained with highly inhomogeneous dose distributions in some cases and very low valley doses [15,17]. However, if needed, a more homogeneous dose distribution could be achieved by narrowing the beams spacing or interlacing several arrays in the tumour [16,41]. In addition, and thanks to the minimal toxicity observed, more aggressive irradiation schemes than the one employed here could be considered in Ne MBRT.

Moreover, there are some indications that heavy ions cans potentiate the immune response in a more effective way than photons, or even proton beams [5]. This might result in a highly effective synergic effect with the potential immune activation in spatially fractionated radiation therapy [42].

The results of this first experiment support the continuation of the exploration of very heavy ions for therapy. A first evaluation of tumour control effectiveness was planned in March 2021, and it is now postponed due to COVID-19 crisis. 

## 5. Conclusions

This manuscript reports the first experimental evidence of the significant gain of normal tissue preservation that is provided by Ne MBRT in comparison with broad beam irradiations. The mice leg’s skin exposed to MBRT showed low local effects, which were mostly characterized by multifocal epidermal hyperplasia, minimal inflammatory reaction, and a decrease in annexal structure numbers. At this timepoint, these local effects were considered to be adaptative and of low clinical significance. In comparison, mice exposed to broad beam exhibited severe side effects. Therefore, Ne MBRT treatments are remarkably better tolerated than broad beam. This result could allow for a renewed use of very heavy ions, whose high linear energy transfer in the target would be unrivalled to overcome tumour hypoxia. 

## Figures and Tables

**Table 1:** Skin damage scores.

Score	Observation
Normal	0
Erythema	1
Dry desquamation	2
Moist desquamation	3
Ulceration	4
Necrosis	5

**Table 2:** Total summarized scores from histology evaluation in each treatment group.

GROUP	Epidermal Hyperplasia	Epidermal Necrosis	Inflammation Infiltration	Dermal Edema	Decrease in Annexa
Control	0.3 ± 0.5	0.0	0.0	0.0	0.0
MBRT	1.0 ± 0.5	0.0	0.5 ± 0.5	0.0	0.5 ± 0.5
BB	1.3 ± 0.7	2.8 ± 1.0	3.3 ± 0.9	2.6 ± 1.2	2.3 ± 0.8

## Data Availability

The data presented in this study are available on request from the corresponding author.

## Supplementary Materials

The following are available online at https://www.mdpi.com/2072-6694/13/6/1356/s1, Table S1 Total summarized scores from histology evaluation in each treatment group.

## Appendix A

Severity scores were assigned Grade 1 to 5.

Grade 1, Minimal	This corresponds to a histopathologic change ranging from inconspicuous to barely noticeable but so minor, small, or infrequent as to warrant no more than the least assignable grade. For multifocal or diffusely distributed lesions, this grade was used for processes where less than approximately 10% of the tissue in an average high-power field was involved.
Grade 2, Slight	This corresponds to a histopathologic change that is a noticeable but not a prominent feature of the tissue. For multifocal or diffusely distributed lesions, this grade was used for processes where between approximately 10% and 25% of the tissue in an average high-power field was involved.
Grade 3, Moderate	This corresponds to a histopathologic change that is a prominent but not a dominant feature of the tissue. For multifocal or diffusely distributed lesions, this grade was used for processes where between approximately 25% and 50% of the tissue in an average high-power field was involved.
Grade 4, Marked	This corresponds to a histopathologic change that is a dominant but not an overwhelming feature of the tissue. For multifocal or diffusely distributed lesions, this grade was used for processes where between approximately 50% and 95% of the tissue in an average high-power field was involved.
Grade 5, Severe	This corresponds to a histopathologic change that is an overwhelming feature of the tissue. For multifocal or diffusely distributed lesions, this grade was used for processes where greater than approximately 95% of the tissue in an average high-power field was involved.
