# Supplementary material for: A Potential Renewed Use of Very Heavy Ions for Therapy: Neon Minibeam Radiation Therapy
Source: Cancers (Basel). 2021 Mar 17;13(6):1356. doi: 10.3390/cancers13061356 (PMC8002595; doi:10.3390/cancers13061356)
Supplement: Supplementary file 1 [file cancers-13-01356-s001.pdf]

Supplemental material

Table S1. Total summarized scores from histology evaluation in each treatment group.

SAMPLE	TREATMENT	EPIDERMAL HYPERPLASIA	EPIDERMAL NECROSIS	INFLAMMATORY INFILTRATION	DERMAL EDEMA	DECREASE IN ANNEXA
1	Control, non irradiated	0	0	0	0	0
2	Control, non irradiated	0	0	0	0	0
3	Control, non irradiated	1	0	0	0	0
4	Irradiated, MBRT	2	0	1	0	0
5	Irradiated, MBRT	1	0	1	0	1
6	Irradiated, MBRT	1	0	0	0	0
7	Irradiated, MBRT	1	0	0	0	0
8	Irradiated, MBRT	1	0	1	0	1
9	Irradiated, MBRT	0	0	0	0	0
10	Irradiated, MBRT	1	0	1	0	1
11	Irradiated, MBRT	1	0	0	0	1
12	Irradiated, BB	1	4	3	4	3
13	Irradiated, BB	2	3	4	4	2
14	Irradiated, BB	2	2	2	2	1
15	Irradiated, BB	2	3	4	1	3
16	Irradiated, BB	2	1	4	4	2
17	Irradiated, BB	2	2	2	2	1
18	Irradiated, BB	0	4	3	2	3
19	Irradiated, BB	1	3	4	2	3
